# A genetic toolkit underlying the queen phenotype in termites with totipotent workers

**DOI:** 10.1038/s41598-024-51772-7

**Published:** 2024-01-26

**Authors:** Silu Lin, Daniel Elsner, Leon Ams, Judith Korb, Rebeca Rosengaus

**Affiliations:** 1 Evolutionary Biology and Ecology, University of Freiburg, 79104 Freiburg, Germany; 2 Research Institute for the Environment and Livelihoods, Charles Darwin University, Casuarina Campus, Darwin, NT 0909 Australia; 3 Department of Marine and Environmental Sciences, Northeastern University, Boston, MA 02115 USA

**Keywords:** Evolution, Zoology

## Abstract

Social insect castes (e.g., queens, workers) are prime examples of phenotypic plasticity (i.e., different phenotypes arising from the same genotype). Yet, the mechanisms that give rise to highly fertile, long-lived queens versus non-reproducing, short-lived workers are not well understood. Recently, a module of co-expressed genes has been identified that characterizes queens compared to workers of the termite *Cryptotermes secundus* (Kalotermitidae): the Queen Central Module (QCM). We tested whether the QCM is shared in termite species, in which queens gradually develop via early larval and late larval instars, the latter functioning as totipotent workers (linear development). Similar as in *C. secundus*, gene expression profiles revealed an enrichment of QCM genes in *Zootermopsis angusticollis* queens, a species from another termite family (Archotermopsidae). The expression of these QCM genes became gradually enriched during development from early larval instars via workers to queens. Thus, our results support the hypothesis of a conserved genetic toolkit that characterizes termite queens with gradual linear development. Our data also imply a strong caste-specific tissue specificity with the QCM signal being restricted to head-prothorax tissues in termite queens. This tissue-specific expression of key aging-related genes might have facilitated the evolution of a long lifespan in termite queens.

## Introduction

Eusocial insects are characterized by reproductive division of labor. Within such insect colonies of termites, ants, bees, or wasps, one or a few individuals specialize in reproduction, while workers (and sometimes soldiers) perform all non-reproductive tasks in the colony, such as foraging, brood care, or colony defense. Associated with this division of labor is a striking increase in the longevity of queens (and kings in termites) compared to worker but also solitary insects^1,2^. How can such a division of labor evolve, and how can different castes develop? Social insect castes are prime examples of phenotypic plasticity, i.e., the expression of different phenotypes from the same genetic background. Within a colony, workers, soldiers, and new reproductives arise due to differential gene expression during ontogeny caused by epigenetic regulations or environmental triggers such as season, differential feeding by nestmates, the presence of predators, or food availability (e.g.,^3–6^).

Within the realm of sociogenomics (*sensu*^7)^, there has been considerable progress in identifying genes and gene networks underlying caste differentiation and caste differences in social Hymenoptera (e.g., ants:^8–10^, bees:^11^, wasps:^12^; reviewed in^13,14^, and references therein). These results revealed that genes and gene networks from solitary insect species were co-opted for caste differentiation (reviewed in^13–15^) and these genes might be part of a genetic toolkit that underlies the evolution of caste^16^. During social evolution, some of these networks have become uncoupled, and their genes heterochronically (i.e., change in the timing of the expression of genes during development over evolutionary time) expressed between castes. Details seem to differ between species and lineages (e.g.,^9,17^). However, there are re-current genes and gene pathways that are associated with nutrient sensing (IIS: insulin/insulin-like growth factor 1 signaling; TOR: target of rapamycin pathways), endocrine (juvenile hormone, JH) regulation, and fecundity (vitellogenin/yolk protein) (e.g.,^15,17–20^). These genes and molecular pathways have been summarized as the TI-J-LiFe (for TOR/IIS-JH-Lifespan/Fecundity) network which underlies life history traits in insects in general^17^. Strikingly, in social insects, genes linked to chemical communication (e.g., cuticular hydrocarbon/CHC synthesis and perception) seem to be important components of the TI-J-LiFe network as well^17,21^.

Compared to social Hymenoptera, little comparative sociogenomic data exist for termites, most concentrating on the development of soldiers in a handful of species (e.g., reviewed in^22–24^), with a few studies on termite reproductives^21,25^. However, transcriptomic studies rarely compared queens with workers and if they did, they used whole bodies which may mask tissue-specific signals (see Discussion) (exception^21^). Termites (infraorder: Isoptera) are ‘social cockroaches’, a monophyletic clade nested within the Blattodea^26^ that evolved eusociality and castes independently from the social Hymenoptera. This different ancestry is reflected in colony composition: Termite colonies are composed of both sexes, with a queen and king heading a colony and with workers that are developmentally immature. Unlike social Hymenoptera, termite workers are immatures, and, therefore, there are no adult workers. A recent study on the drywood termite *Cryptotermes secundus* (Kalotermitidae) identified a module of 288 co-expressed genes from head plus prothorax tissue, the queen central module (QCM), which characterizes queens^21^ (Figure S1). The QCM comprises central molecular pathways that underlie a queen phenotype. It has a strong neuro-endocrine signal indicative of high JH titers in line with an upregulation of fecundity-related genes, such as vitellogenins (Figure S1). The QCM also included signs of an upregulation of the IIS pathway as well as signals of chemical communication, similar to those in many social Hymenoptera^15,17^.

In the current study, we first aimed to test whether the QCM exists in the Archotermopsidae as well, a termite family with a more basal phylogenetic position than the Kalotermitidae but with similar life style and caste development. Like *C. secundus*, *Z. angusticollis* is a wood-dwelling termite that nests in a piece of wood that serves both as food and shelter (one-piece nester *sensu*^27^) without ever leaving the nest to forage outside. As typical for this life type, both species have a low level of social complexity and a linear development in which early larval instars develop into older larval instars that function as workers from which all reproductives develop (reviewed in^28^). *Z. angusticollis* larvae initiate labour, such as brood care, from the third instar onwards. Accordingly, individuals at the third instar or older are designated as workers. As in all termites, *Z. angusticollis* workers are always immatures; in wood-dwelling termite species with a linear development, they can transition into adults and with doing this they become reproductives. Given that these two species have a shared linear developmental pathway but belong to different families, we can test the existence of a shared genetic toolkit that characterizes queens, without confounding factors that might arise when caste development differs. Termites not belonging to the wood-dwelling life type have a bifurcated development; i.e., there is a split into two developmental lines, one leading to wingless individuals (apterous line, mainly workers and soldiers) and the other to winged reproductives (nymphal line)^28–30^. This bifurcation of development means changes in the underlying developmental program, which makes queen-worker comparisons complicated across species with different developmental trajectories.

To test whether we can detect a QCM signal in *Z. angusticollis*, we generated and compared transcriptomes for queens and workers. We also tested how workers differed in gene expression from early instar larvae, from which they developed, by comparing transcriptomes of early instar larvae (1^st^ and 2^nd^ instars, which do not provide labor, hereafter larvae) from worker instars (≥ 3^rd^ instar larvae, which do perform labor in the nest, hereafter workers)^31^.

Transcriptomes were generated for two tissues: head plus prothorax (hereafter ‘head’ for simplicity) and abdomen without gut (hereafter ‘abdomen’ for simplicity). The former tissues correspond to those used in the *C. secundus* study^21^, including neuro-endocrine signals. Note, the tissue of juvenile hormone (JH) biosynthesis, the corpora allata that are located at the posterior end of the brain, can be lost during dissection, when using head only. Hence, we used head plus prothorax. The abdominal tissue extended the analyses to reveal, for example, stronger fecundity and fat body related signals.

## Results

In total, we found 262 homologs of the 288 *C. secundus* QCM genes in the analysis for *Z. angusticollis*.

### Gene expression patterns characterizing queens compared to workers

#### Heads

##### Differentially expressed genes (DEGs)

In heads, 479 genes were more highly expressed in workers than queens (Data S3) and they were mainly characterized by genes related to cuticle proteins and transcription factors. There were four homologs to genes of the *C. secundus* QCM (for simplicity, hereafter ‘QCM genes’) among them, and QCM genes were not enriched (Fisher’s exact test: *P* = 0.117) (Table S2: Enrichment results).

**Figure 1:**
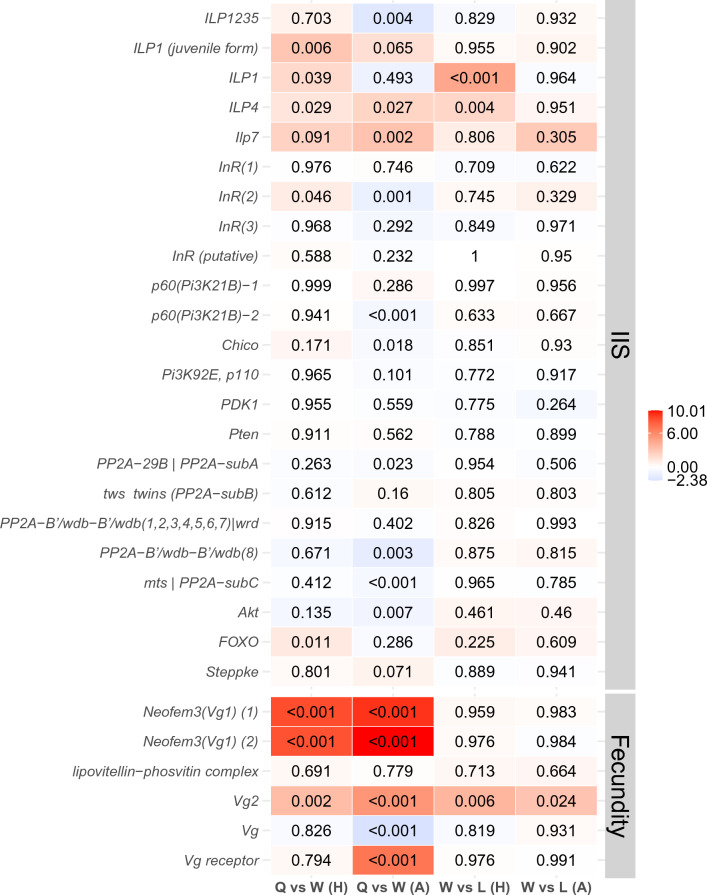
Results of the differential gene expression analyses for genes from the IIS and fecundity-related pathways. Shown are QCM genes. Each row represents a gene. Column 1 shows the results for the queen-worker comparison of head samples, and column 2 those of the abdomen samples. Column 3 and 4 represent the results for the worker-larvae comparisons for heads and abdomens, respectively. The color bar reflects the log2 fold change (LFC) in gene expression, with red indicating a higher expression of the first group compared to the second group (e.g., in column 1, a higher expression in queens than workers) and blue vice versa. The value in each cell shows the adjusted *P* value. *Q* queens, *W* workers, *H* heads, *A* abdomens, *L* larvae.

**Figure 2:**
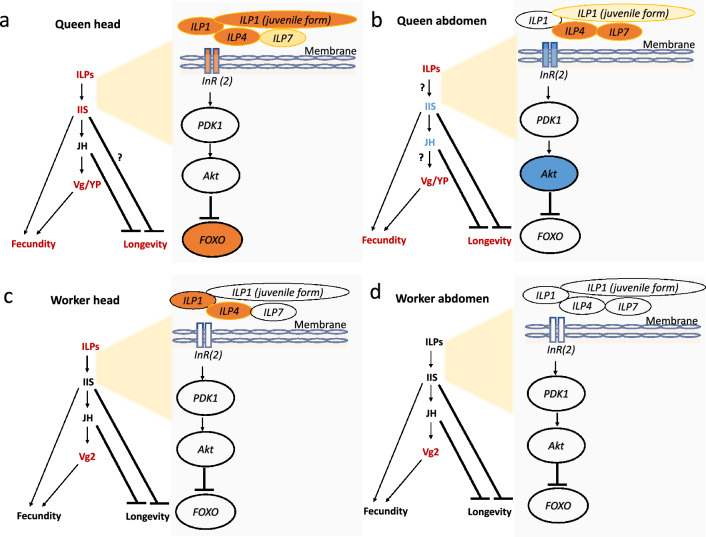
Caste- and tissue-specific gene expression patterns with a focus on genes related to IIS, JH, and Vg/YP. Shown are expression patterns characterizing (**a**) queen head compared to worker head, (**b**) queen abdomen compared to worker abdomen, (**c**) worker head compared to larval head, and (**d**) worker abdomen compared to larval abdomen. Solid arrows denote activation, while stop bars indicate repression. Question marks highlight unusual gene expression patterns contradicting expectations. Red and orange colors signify upregulation, blue indicates downregulation, and golden color represents upregulation by trend. For details, see main text. Relationships between genes are drawn based on previous studies^19,50^.

On the other hand, 251 DEGs were more highly expressed in queens compared to workers (Data S3), among them sixteen were QCM genes (Data S5). Thus, QCM genes were significantly enriched among the DEGs characterizing *Z. angusticollis* queens (Fisher’s exact test: *P* < 0.001) (Table S2: Enrichment results). Accordingly, many QCM genes, and associated TI-J-LiFe genes, characterized queen DEGs compared to workers. There was a strong fecundity signal with three fecundity-related termite *Vgs* (Fig. 1). In addition, the upper part of the IIS pathway seems to be upregulated in queens compared to workers, as an *InR* and three ILPs genes are more highly expressed in the former (Figs. 1, 2a). However, genes further downstream of the IIS pathway were not significantly higher expressed in queens compared to workers (Fig. 1). Interestingly, the longevity gene *FOXO*, which is typically negatively affected by active IIS signaling^32,33^, was upregulated in queens (Figs. 1, 2a).

**Figure 3:**
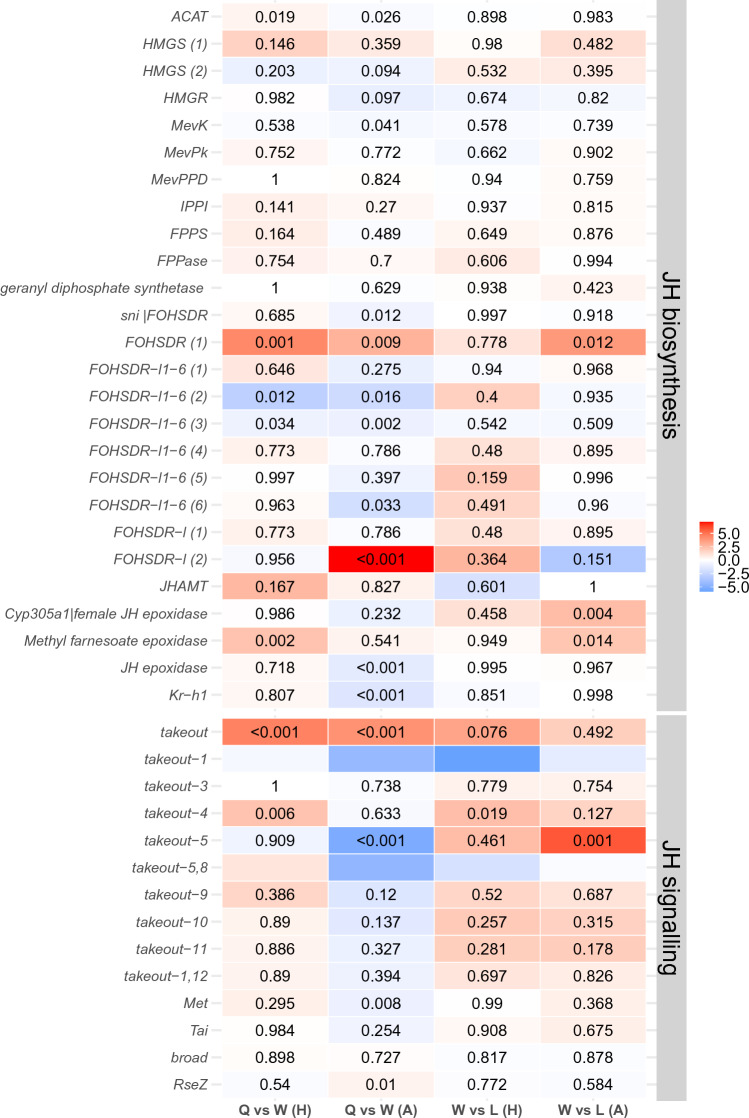
Results of the differentially expressed gene analyses on JH-related genes for the queen-worker comparison (column 1 for head tissue and column 2 for abdomen tissue) and for the worker-larvae comparison (column 3 for head tissue and column 4 for abdomen tissue). Shown are QCM genes. For more information, see Fig. 1. *Q* queens, *W* workers, *H* heads, *A* abdomens, *L* larvae.

The endocrine JH signal was ambiguous. A farnesol dehydrogenase, *FOHSDR (1)* (supposedly involved in JH biosynthesis^34^), the *methyl farnesoate epoxidase* (also known as *CYP15A1_7*) (catalysing the conversion of methyl farnesoate to juvenile hormone III in a cockroach^35^), and two *takeout* genes (typically encoding JH binding proteins) were higher expressed in queens than workers (Figs. 2a, 3). However, the early response gene of JH signaling *Kr-h1* (*Kruppel homolog 1*), was not differentially expressed. Furthermore, as is typical for the QCM, several genes related to trehalose metabolism (e.g., three *TRET* genes) as well as many genes putatively involved in CHC biosynthesis (e.g., one desaturase, three elongases, and one fatty acyl-CoA reductase), were found among the queen DEGs (Figure S2, S3). In addition, some immune defense genes (lysozyme-related genes and toll-like receptor) were also upregulated in queens (Data S3).

##### Network analyses

The gene co-expression network analysis revealed nine modules that were positively associated with workers compared to queens (Data S6). Module “*yellow4*” contained four QCM homologs and it was enriched with QCM genes (Fisher’s exact test: *P* = 0.035). However, the four QCM homologues (Znev_02208, Znev_10922, Znev_12889, and Znev_18213) are not TI-J-LiFe genes.

Seven modules were significantly associated with queens compared to workers (Data S7). QCM genes were significantly enriched in the modules *red*, *darkolivegreen*, *turquoise*, and *darkred* (Table S3). Yet, other modules also contained QCM genes, although they were not significantly enriched (Tables S3). The module *red* can be functionally characterized as a fecundity-chemical communication module. It contained the three fecundity-related termite *Vgs*, one gene identified as insulin-like growth factor, five *takeout* genes, and several genes potentially related to CHC production and perception (desaturase, elongase, odorant receptor, and odorant-binding protein) (Data S7). The queen module *turquoise* comprised a striking combination of co-expressed genes. It included genes putatively related to JH biosynthesis (farnesol dehydrogenases, *FOHSDR-l1-6 (1), FOHSDR-l1-6 (4), HMGS (1), Jheh1,2*), several IIS genes (among them *ILP1*), and the anti-aging gene *FOXO*. This apparent concordant co-expression of some IIS genes with *FOXO* is striking. Typically, *FOXO* is inhibited by an upregulation of the IIS pathway in animals, and this negative association seems to be a major molecular cause of the common trade-off between longevity and fecundity^32,33^. A positive association between IIS and *FOXO*, as indicated by the queen module *turquoise*, might contribute to explaining the absence of this trade-off in queens. However, the phosphorylation status of FOXO needs to be studied to determine its functional activity. In addition, the *turquoise* module included several genes putatively related to CHC production (*acsbg2*, elongase, and fatty acyl-CoA reductase) and genes related to trehalose metabolism (*TRET*), characteristic for the QCM. It was the module most similar to the *C. secundus* QCM.

#### Abdomen

##### DEGs

In the abdomen, 2,715 DEGs were more highly expressed in workers than queens; many of them were cuticle proteins (Data S3). Surprisingly, among the DEGs, there were 77 QCM homologs, and they were enriched with QCM genes (Fisher’s exact test: *P* < 0.001) (Table S2).

2,653 DEGs were more highly expressed in queens than in workers (Data S3), among them 29 QCM genes. In contrast to the head, QCMs were less likely to occur among the queen DEGs in the abdomen than expected by chance (Fisher’s exact test: *P* = 0.001) (Table S2). This might be due to the use of a different tissue, as the original QCM was identified from the heads of *C. secundus*.

The importance of tissue specificity is supported by the gene-specific analyses concentrating on QCM and TI-J-LiFe genes (Figs. 1, 2b, 3). Only fecundity-related *Vgs* and *ILP4* and *ILP7* were consistently more highly expressed in queens than workers across body parts (Figs. 1, 2a,b). Many IIS pathway genes (e.g., *InR2*, *chico*, and *Akt*) as well as JH-related genes, including the JH-signaling gene *Kr-h1*, were more highly expressed in workers’ abdomens (Figs. 1, 2b, 3). This suggests that in the abdomen both pathways are downregulated in queens, in contrast to the head (Fig. 2a,b).

##### Network analyses

The network analysis revealed ten modules that were positively associated with workers (Data S8). Among them, four modules (*midnightblue*, *brown*, *yellow*, *salmon*) were enriched with QCM genes (statistical results see Table S4). These modules contained genes that are supposedly involved in JH synthesis (e.g., *JH epoxidase, HMGR, FOHSDR-l1-6 (3)*)*,* JH signalling (*Kr-h1*, several *takeout* genes) and CHC production (elongases, desaturases) and perception (ORs).

Sixteen network modules were significantly positively associated with queens compared to workers (Data S9). None of these modules were enriched with QCM genes (Table S3). The module *royalblue* contained two fecundity-related *Vgs* and many genes related to histones and histone modification including *Neofem9*, which is a Histone H2A, identified to be queen-specifically expressed in the termite *Cryptotermes cynocephalus*^36^.

### Gene expression patterns characterizing workers compared to larvae

#### Head

##### DEGs

In the heads, 34 DEGs were more highly expressed in larvae than workers. Among them, there was one QCM homolog and QCM genes were not enriched (Fisher’s exact test: *P* = 0.469). On the other hand, 160 DEGs were more highly expressed in workers than larvae. These worker DEGs contained 12 QCM homologs and were enriched with QCM genes (Fisher’s exact test: *P* < 0.001). This implies that workers are more similar to queens than larvae.

The similarity between queens and workers (compared to larvae) is reflected at a gene-specific level. Among the worker DEGs was one of the three fecundity-related vitellogenin, *Vg2*, (Figs. 1, 2c) as well as *ILP1* and *ILP4* and several *takeout* genes (Figs. 1, 2c, 3). However, there were no signs of differential expression between workers and larvae of other IIS or JH-related genes (Figs. 1, 2c, 3). Reflecting the QCM signal, some metabolic genes (two *TRET* genes, trehalose) and genes putatively related to chemical communication (delta(11)Desaturase, elongase, *acsbg2*; OBPs) were detected (Figure S2, Figure S3).

##### Network analyses

For larvae heads, the network analyses revealed two modules (Data S10). The module *thistle3* contained two genes related to histone modification. The module *green* contained several gustatory and odorant receptors and many genes related to transcription regulation (e.g., RNA splicing). With four QCM homologs, it might be enriched with QCM genes (Fisher’s exact test: *P* = 0.050, Table S5). Yet, only one of the four QCM homologs is well annotated: regucalcin, which is involved in sexual reproduction and diapause in *Drosophila* but is also expressed in larval somatic tissues^37–40^.

Four modules characterized workers compared to larvae (Data S11), all were enriched for QCM genes (for statistical results, see Table S6). The module *paleturquoise* comprised co-expressed genes that reflected the similarity of workers with queens. It contained *Vg2*, *ILP1* and *ILP4*, and some of the genes related to chemical communication. Although there was no apparent JH signal among the worker DEGs, the worker module *turquoise* was strongly related to JH biosynthesis and regulation. The co-occurrence of a geranyl diphosphate synthetase, three *farnesol dehydrogenase-like* genes (one of them is a homolog of *FOHSDR-l1-6 (4)* from *C. secundus*), and the *methyl farnesoate epoxidase* (also known as *CYP15A1_7*) implies that JH biosynthesis genes are co-expressed. Furthermore, a JH epoxide hydrolase and eight takeout genes were also in this module as well as several genes potentially linked to chemical communication (several ORs, fatty acyl-CoA reductase, desaturase, elongases).

#### Abdomen

##### DEGs

In the abdomen, 62 DEGs were more highly expressed in larvae than workers; there was no QCM homologs among these DEGs. QCM genes were not enriched (Fisher’s exact test: *P* = 0.632). These were characterized by genes that have been associated with development (*sonic hedgehog*, *ecdysone induced 78C*, *abdominal B*)*.*

In comparison*,* 118 DEGs were more highly expressed in workers than larvae, and they were significantly enriched for QCM genes (Fisher’s exact test: *P* < 0.001). The signal was similar to that of the head in that it contained the same fecundity-related *Vg2* (Figs. 1, 2d). However, in contrast to the head, two putative JH biosynthesis genes (the farnesol dehydrogenase *FOHSDR (1)*, the *methyl farnesoate epoxidase* (also known as *CYP15A1_7*), and *CYP305a1/ female JH epoxidase* were higher expressed in workers than larvae. In addition, several *takeout* genes, were among the worker DEGs (Fig. 3).

##### Network analyses

For larvae abdomens, the network analysis revealed two modules (Data S12), each with one QCM homolog and no enrichment of QCM genes (Table S5). The module *palevioletred2* contained several genes related to histones and growth. The module *cyan* contained many genes related to histones and transcriptional splicing.

For workers, one module, *black* (Data S13), was uncovered that was significantly associated with workers, and it was significantly enriched with QCM genes (Fisher’s exact test: *P* = 0.011). It contained some of the worker DEGs, including *Vg2* and the *methyl farnesoate epoxidase* (*CYP 15A1*_7).

## Discussion

### Is there a QCM signal in *Z. angusticollis*?

QCM genes that characterized gene expression in the head (plus prothorax) of *C. secundus* queens were similarly active in the head (plus prothorax) of *Z. angusticollis* queens. This suggests that the QCM is a conserved toolkit that gives rise to the queen phenotype in these two species from different families with shared linear development. Furthermore, the comparison of workers and larvae revealed a QCM gene enrichment in the workers. As young larvae progress into older worker instars, from which reproductives eventually emerge, the recurring QCM signal suggests a gradual increase in the expression of QCM genes (i.e., genes typical of queens) during development. This would reflect the gradual development of a hemimetabolous insect, which differs fundamentally from the holometabolous social Hymenoptera, in which a major re-structuring occurs during the pupal stage. In line, signals of queen differentiation increase drastically when pupal metamorphosis starts in ants^41^. Future research can test the hypothesis that the molecular signatures distinguishing reproductives in termites become apparent gradually, at least in wood-dwelling termite species with a linear development. This is expected to differ in termite species with a bifurcated development of an apterous and a nymphal line, in which workers that develop along the apterous line lose the potency to become winged sexuals^28,42^.

Furthermore, the QCM signal appears to be consistent across sexes. Given the limitations of sex differentiation based on morphology, we sampled larvae and workers randomly, encompassing both sexes. Yet, when examining the PCA, the distinctions between sexes appeared negligible as all workers and all larvae formed cohesive clusters (Figure S4-S8).

### Fecundity related genes: vitellogenins

The QCM signal is not driven by the three fecundity-related Vg genes. They were more highly expressed in the abdomen of queens than workers. Yet there was no enrichment for QCM genes in the queens’ abdomen; in fact, QCM genes were even less common than expected.

The Vg signal was present in both body parts, in the queen (compared to worker) DEGs (Figs. 1, 2a,b) and worker DEGs (compared to larvae) (Figs. 1, 2c,d). In queens, all three fecundity-related Vgs (*Neofem3 (Vg1) (1)*, *Neofem3 (Vg1) (2)*, and *Vg2*) were overexpressed, while only *Vg2* was more highly expressed in workers (compared to larvae) (Fig. 2). This implies that the two *Vg1 in Z. angusticollis*, which seem to be the result of a gene duplication of *Neofem3* of *C. secundus*^43^, are reproductive-specific. This is in line with results for *C. secundus*, in which *Neofem3* is also only upregulated in reproductives and across all body parts^44^. Vg genes are mainly expressed in fat bodies, which occur across a termite’s body^45^. In the head of queens and in the non-reproducing workers, these three Vg genes may function as storage proteins and may have additional functions such as serving as anti-oxidants like in social Hymenoptera (e.g.,^46^). In the abdomen of queens, the concordantly high expression of the *Vg receptor* (Fig. 1) reflects their role as egg yolk precursors in egg production (e.g.,^3^).

In addition, there was a fourth Vg in *Z. angusticollis*, *Vg*. The expression of this gene in the abdomen seems to characterize immatures as it was overexpressed in workers compared to queens with no difference between workers and larvae (Fig. 1). *Vg* probably functions as a storage protein or has other non-reproductive functions as in social Hymenoptera (e.g.,^46^) and termites^47^.

### Distinct body-part-specific gene expression across castes

There was a strong body-part specificity of QCM gene expression. A body-part specificity of gene expression is not surprising as different tissues have different functions. Yet, the specific pattern is insightful. Only in the head (plus prothorax) tissues were QCM genes enriched among queen DEGs (compared to workers), while they were less common than expected in the abdomen and enriched among worker DEGs (compared to queens). Focusing on the TI-J-LiFe network reveals that this pattern is largely due to a lower expression of genes from the JH- and IIS-pathways in the abdomen of queens (compared to workers) (Figs. 1, 2a,b, 3). It is not surprising to detect an upregulation of late JH biosynthesis genes (like *methyl-farnesoate epoxidase*; Fig. 3) in queens heads (plus prothorax) (compared to workers) but not in their abdomen. The production sites of JH, the corpora allata, are in the former and termite queens are characterized by high JH production^48,49^. However, it is striking that *Kr-h1*, the early response gene of JH signaling, is upregulated in workers’ abdomen compared to queens (Fig. 3), though we would expect it to be higher in queens if they have higher JH titers, or at least not different as *Kr-h1* in the head it is not differentially expressed. Several IIS genes (*ILP1235, p60(PiK21b)-*2, *PP2A-B’, PP2A-subC*), including the IIS ‘exit’ gene *Akt*, show a similar pattern as *Kr-h1*, implicating a specific lower IIS activity in the queens’ abdomen (Fig. 2b). As reduced IIS signaling is associated with prolonged longevity in multiple organisms, like the fruit fly *Drosophila melanogaster* and the nematode *Caenorhabditis elegans*^50^, the abdomen-specific down-regulation in queens might contribute to the long lifespan of termite queens. This is supported by a recent experimental study, in which *C. secundus* queens that received a protein-enriched diet had increased survival rates compared to those that did not^51^. Associated with increased queen survival, a similar downregulation of IIS genes (including *Akt*) and *Kr-h1* was observed in abdominal fat bodies, while they were unaffected in the head (plus prothorax)^51^.

Comparing workers and larvae, we did not see a similar body-part specific lower expression of IIS- and JH-genes (Figs. 1, 2c,d, 3). This may suggest a downregulation of IIS and JH activity in the abdomen after queen differentiation. As both of these pathways have been associated with aging in many animals^50,52^, this body-part specific expression may contribute to the high longevity of *Z. angusticollis* queens of around six years^2^.

Unfortunately, it is difficult to compare our results in detail with other transcriptome studies that included queens, as they compared queens across age classes- rather than with workers^53–55^ and / or they used whole body transcriptomes^56,57^. Whole body transcriptomes are problematic because tissue-specific signals may cancel out each other, as is shown in our study.

The QCM is specific for the head (plus prothorax) and failure to choose the appropriate tissues may explain why other social insects studied could not identify a QCM yet^21^. For example, using only the head can lead to a loss of the corpora allata, the gland of JH biosynthesis, as dissections in our laboratory have shown. At a very broad scale, there is an IIS (and ILP) signal in termite queens^53,55,57^, which aligns with our results and that of social insects in general^15,17^. However, such superficial comparisons provide little insight as this is the default to be expected for reproducing female insects. Only detailed studies, which distinguish at least between relevant body-parts and which analyze (relevant) pathways in detail, will help to figure out what makes termite queens special. Allometric tissue differences between castes (i.e., tissue differences that do not scale proportional with size) could bias gene expression differences. They might also partly influence the gene expression results of our study. Yet this effect should be minor, as obvious allometries are unknown for our species. The application of advanced technologies like single-cell sequencing could offer an ultimate solution, particularly as these technologies become more widely available.

## Conclusion

We showed that the QCM that characterize *C. secundus* queens is also typical for *Z. angusticollis* queens. This implies a conserved genetic toolkit that gives rise to the queen phenotype across two termite families with a linear development. Furthermore, QCM genes seem to become increasing expressed during the development from larvae via workers to queens in head plus prothorax tissues. Based on these results, we hypothesize that *a head-prothorax-specific QCM* signal is shared, at least in termites of low social complexity characterized by totipotent workers and a linear development.

Surprisingly for the *abdomen,* QCM genes were enriched in workers compared to queens. This signal is largely driven by a high expression of JH- and IIS-related genes in workers. This result stresses the importance of tissue specificity to reveal the QCM signal. In addition, the tissue-specific high expression of JH- and IIS-genes in queens, limited to the head-prothorax tissue but not in the abdomen, might contribute to the long lifespan of *Z. angusticolli*s queens, as a high expression of both pathways has been linked with aging in many animals.

## Materials and methods

### Termite collection and maintenance

Six mature *Zootermopsis angusticollis* colonies were collected from the Redwood East Bay Regional Park in Oakland, California. The colonies with their original wood/nest material were placed inside covered plastic tubs and flown to Northeastern University under an USDA permit (P526P-17-03817). The study was conducted in accordance with the Nagoya protocol and all authors followed ARRIVE guidelines. The colonies were kept in the dark at 23 to 25 °C. They were sprayed with water twice a week to maintain a 60% relative humidity. Birchwood was added as needed to provide termites with additional food/nesting resources.

### Establishment of incipient colonies and maintenance

*Z. angusticollis* alates (winged individuals) were collected from mature colonies after molting. They were sexed under a dissecting microscope and paired inside plastic Petri dishes (60 mm diameter X 15 mm height) lined with moistened (300 µL sterile water) Whatman # 1 filter paper discs. Each pair also received ~ 2.5 mg of birch wood to build a copularium (i.e., mating chamber). Only heavily sclerotized alates with intact wings were used. This ensured that paired alates were similarly motivated (both physiologically and behaviorally) to mate^58–60^. These alates were virgins, as *Z. angusticollis* alates complete reproductive maturation only when separated from their parental nest^61^, and copulation takes place only after the pair has constructed a copularium^62^. The Petri dishes were stacked inside clear, covered plastic boxes lined with wet paper towels to maintain high humidity (~ 90% relative humidity). Water and birch wood chips were added as needed. We originally set up hundreds of incipient colonies. Yet, *Z. angusticollis* has a high failure rate during the early stages of colony foundation (~ 60% mortality^58,63^). Thus, six months post-pairing, we had a total of 42 intact incipient colonies, 25 headed by nestmate reproductives (i.e., originating from the same parental nest, considered inbred), and 17 headed by non-nestmate pairs (originating from different parental colonies, considered outbred). All colonies were newly established and had no soldiers. In the end, we used seven incipient colonies (2 headed by non-nestmates and 5 by nestmate reproductives) to generate transcriptome data (Individual details are listed in Data S1). As we did not see any obvious differences in the gene expression profiles between out- and inbred colonies, we combined both data sets (see Figure S4-S6).

### Collection of termites and storage

Given that the incipient colonies were established on different days, we controlled for the age of the incipient colony by standardizing the number of days elapsed since pairing to about 180 days post-pairing. At this point, the incipient colonies comprised the queen, the king, and a variable number of eggs, larvae, and workers^63^. *Z. angusticollis* queens have a lifespan of around six years^2^.

The queens were cold immobilized and decapitated under a dissecting microscope (~ 40X). Each individual (head + prothorax and abdomen) was then placed inside a PCR tube containing 200 µL of cold RNAlater and immediately stored at 4 °C for 24 h and then frozen under − 80 °C. The other individuals were similarly treated (dissected, submerged in 200 µL RNAlater, and frozen), except that all individuals of the same instar/incipient colony were pooled in the same tube. In total, we obtained 58 individual samples. We selected 36 samples with the best RNA quality for sequencing, making sure that we had each caste per colony. Subsequently, the tubes were kept frozen in a − 80 °C freezer until shipped to Freiburg (Germany) on dry ice, where they were stored at − 20 °C until extraction.

### Generation of transcriptomes

Total RNA was extracted from both tissues of single individuals (no pooling of samples) using a protocol optimized for termites as described elsewhere^21^. This protocol allowed us to obtain enough high-quality RNA for body parts of single individuals. In short, the tissue was homogenized with peqGOLD TriFast™ (Peqlab) for 2–3 min. Then, we added chloroform to separate the aqueous phase and subsequently, nuclease-free glycogen (5 mg/ml) and cold isopropanol (Ambion) to precipitate the total RNA. We then washed the pellet using 75% ethanol and centrifuged the samples for 5 min at 4 °C, 8500 rpm. The washing step was repeated three times. After washing, the pellet was dissolved in nuclease-free water and kept at 4 °C for at least 3 h. DNA digestion was done using DNase I recombinant (Roche) and EDTA (Sigma-Aldrich). Samples were stored at − 80 °C until sending them to BGI (Hong Kong) on dry ice. RNA quality assessment and library preparation were done by BGI with the TruSeq RNA Library Prep Kit v2 (Illumina). HiSeq Transcriptome Sequencing was done on an Illumina HiSeq Xten platform (150-bp paired-end reads), resulting in ~ 4 Gigabases of raw data and 30 to 50 million reads per sample.

#### Quality control, trimming, mapping

We checked the quality of the Illumina raw reads using FastQC v. 0.11.5 and trimmed reads with Trimmomatic v.0.39, removing adapter sequences and keeping only paired-end reads with a minimum length of 120 bp^64,65^. As there is no sequenced genome of *Zootermopsis angusticollis* available, we mapped the trimmed reads with hisat2 to the genome of the sister species *Zootermopsis nevadensis*, v. 2.2^43^. While other strategies such as Trinity-based de novo transcriptome assembly could have been employed, this was not a good alternative in our study, given our focus on gene expression, rather than transcript variants and alternative splicing. A Trinity-based trial revealed 678,095 transcripts that, for instance, could not be unambiguously associated with the QCM genes. Therefore, we mapped the reads to the genome of the sister species. We discarded two samples (LG1OW1H and LG3YW1H) because of low mapping rates of 14.5% and 32.9%, respectively (Data S1). We counted the reads against the reference genome using HTSeq count with the mode “union.” In the end, we had a sample size of six for each investigated caste and tissue, except for the abdomen of workers and larvae, for which we had five replicates.

#### Annotation

To identify genes, we aligned all peptide sequences from the *Z. nevadensis* official gene set v.2.2 to the non-redundant protein database (obtained July 16, 2021) using NCBI Blast + v2.10.0^66^. We set a minimum e-value of 1e^-5^. For genes where the first match was ‘hypothetical’ or ‘unknown,’ we kept the next match if it fulfilled the 1e^-5^ e-value cutoff (Data S2: gene annotation list). Using the annotation results, we further identified all TI-J-LiFe genes (Data S2: TI-J-LiFe genes). Additionally, we used InterProScan v. 5.53–87.0^67^ applying the analyses Pfam, PANTHER, CDD, Gene3D, HAMAP, PIRSF, PRINTS, and SMART to obtain Gene Ontology (GO) annotation. We collected all GO terms for each gene.

#### Differential gene expression analysis

Differentially expressed genes (DEGs) were identified using DESeq2, v. 1.32,0, in R 4.1.1^68^). Gene expression data were first normalized using the varianceStabilizingTransformation() function from DESeq2. The normalized data were used to perform DEG analysis using the DESeq() function. *P* values were calculated using the Wald test and corrected for multiple testing with the false discovery rate (FDR) approach^69^. We defined genes as DEGs if their corrected *P* values were smaller than 0.05.

DEG analyses were performed separately for both tissues (head or abdomen) and for the following comparisons: (i) queens versus workers (Data S3) and (ii) workers versus larvae (Data S4). We also did a Principal Component Analysis (PCA) using the 500 genes with the greatest variance, separately for the abdomen (Figure S4) and head (Figure S6), as well as for both tissues combined (Figure S7, S8).

### Network analysis

Weighted gene co-expression analyses (WGCNA)^70,71^ were applied to identify networks of co-expressed genes (modules) that characterize the phenotypes (‘traits’ in WGCNA terms) of (i) queens compared to workers and (ii) workers compared to larvae. We did these analyses separately for the head and abdomen.

#### Assessing data quality for WGCNA

We first normalized the gene count data using the rlogTransformation() function from DESeq2 (version 1.32.0). To guarantee high data quality, we then performed hierarchical clustering to check for outliers using the function hclust() in the R package flashClust() (version 1.1.2). No outliers needed to be removed as samples of the same phenotype clustered together. Genes with more than 50% missing values and zero variance were removed iteratively (Table S1) using the goodSamplesGenes() from the WGCNA package (version 1.70.3).

#### WGCNA

Normalized gene counts of good quality were used as input to construct a signed adjacency matrix with the most suitable soft-threshold powers, estimated separately for each analysis (Table S1). Average linkage hierarchical clustering analyses were performed on an adjacency-based dissimilarity matrix using the hclust() function. Modules (minimum 30 genes) were detected using the cutreeDynamic() function from the package dynamicTreeCut (version 1.63.1). Eigengenes of the modules were determined using the moduleEigengenes() function. We then calculated module-trait (i.e., phenotype) associations using the cor() function. The asymptotic *P* values for the Student T-test of all module-trait associations were calculated using the corPvalueStudent() function. The hub gene of each module (i.e., the gene with the highest connectivity within the module) was identified with the chooseTopHubInEachModule() function. Gene–trait associations and their corresponding Student *P* values were calculated using the corAndPvalue function.

### Gene ontology (GO) enrichment analysis

Using the GO annotations obtained from InterProScan, we tested whether GO terms were overrepresented in the genes that were highly expressed in (i) queens compared to workers, and (ii) workers compared to larvae. The GO enrichment analysis was performed for all DEGs and striking WGCNA modules. The background for the enrichment analysis was all genes with GO annotations from the whole genome. We applied Fisher's exact tests, as implemented in the R package TopGO v. 2.44.0^72^, to determine the significance level. The results of GO analysis for DEGs are provided in the Supplementary materials.

### QCM enrichment analysis

We tested whether the QCM genes reported in *C. secundus*^21^ were enriched among the DEGs and modules that characterized queens compared to workers (i.e., queen modules) and those that characterized workers compared to larvae. To obtain homologs of *C. secundus* genes in *Z. nevadensis*, we used all amino acid sequences from the *C. secundus* genome^73^ and performed a local BLAST search against all amino acid sequences from the *Z. nevadensis* genome (version 2.2: http://termitegenome.org/?q=consortium_datasets60), using BLASTP (version 2.11.0). We took the best hits (i.e., the hit with the lowest e-value and the highest bit score; at least e-value 1e^-5^). The BLAST search yielded 28,397 hits and 10,420 unique *Z. nevadensis* homologs, of which 262 were QCM homologs. We then used Fisher’s exact test to test whether the occurrence of QCM homologs in the DEGs/modules was significantly higher/lower than expected (i.e., higher/lower than the occurrence of QCM homologs among all expressed genes).

### Supplementary Information

Supplementary Information.

Supplementary Information.

Supplementary Information.

Supplementary Information.

Supplementary Information.

Supplementary Information.

Supplementary Information.

Supplementary Information.

Supplementary Information.

Supplementary Information.

Supplementary Information.

Supplementary Information.

Supplementary Information.

Supplementary Information.

## Data Availability

Raw sequence reads are deposited at the National Center for Biotechnology Information (NCBI) SRA (BioProject PRJNA950229).

## References

[1] Keller L, Genoud M (1997). Extraordinary lifespans in ants: A test of evolutionary theories of ageing. Nature.

[2] Korb, J. & Thorne, B. Sociality in Termites in *Comparative social evolution* 124–157 (2017).

[3] Engels, W. *Social Insects: An Evolutionary Approach to Castes and Reproduction. Social Insects* (Springer Berlin Heidelberg, 1990). 10.1126/science.250.4985.1283 17829216

[4] Lenz, M. Food Resources, Colony Growth and Caste Development in Wood-feeding Termites in *Nourishment and evolution in insect societies* (eds. Hunt, H. J. & Nalepa, A. C.) 159–209 (WestviewPress, 1994).

[5] Korb J, Katrantzis S (2004). Influence of environmental conditions on the expression of the sexual dispersal phenotype in a lower termite: implications for the evolution of workers in termites. Evol. Dev..

[6] Okwaro LA, Korb J (2023). Epigenetic regulation and division of labor in social insects. Curr. Opin. Insect Sci..

[7] Robinson GE, Grozinger CM, Whitfield CW (2005). Sociogenomics: Social life in molecular terms. Nat. Rev. Genet..

[8] Qiu B (2018). Towards reconstructing the ancestral brain gene-network regulating caste differentiation in ants. Nat. Ecol. Evol..

[9] Warner MR, Qiu L, Holmes MJ, Mikheyev AS, Linksvayer TA (2019). Convergent eusocial evolution is based on a shared reproductive groundplan plus lineage-specific plastic genes. Nat. Commun..

[10] Nagel M (2020). The gene expression network regulating queen brain remodeling after insemination and its parallel use in ants with reproductive workers. Sci. Adv..

[11] Kapheim KM (2015). Social evolution. Genomic signatures of evolutionary transitions from solitary to group living. Science.

[12] Patalano S (2015). Molecular signatures of plastic phenotypes in two eusocial insect species with simple societies. Proc. Natl. Acad. Sci. USA.

[13] Rehan SM, Toth AL (2015). Climbing the social ladder: the molecular evolution of sociality. Trends. Ecol. Evol..

[14] Toth AL, Rehan SM (2017). Molecular evolution of insect sociality: An eco-evo-devo perspective. Annu. Rev. Entomol..

[15] Weitekamp CA, Libbrecht R, Keller L (2017). Genetics and evolution of social behavior in insects. Annu. Rev. Genet..

[16] Toth AL, Robinson GE (2007). Evo-devo and the evolution of social behavior. Trends Genet..

[17] Korb, J. *et al.* Comparative transcriptomic analysis of the mechanisms underpinning ageing and fecundity in social insects. *Philos. Trans. R. Soc. Lond. B Biol. Sci.* **376**, 20190728 (2021). 10.1098/rstb.2019.0728 PMC7938167 33678016

[18] Corona M (2007). Vitellogenin, juvenile hormone, insulin signaling, and queen honey bee longevity. Proc. Natl. Acad. Sci. USA.

[19] Rodrigues MA, Flatt T (2016). Endocrine uncoupling of the trade-off between reproduction and somatic maintenance in eusocial insects. Curr. Opin. Insect. Sci..

[20] Kapheim KM (2017). Nutritional, endocrine, and social influences on reproductive physiology at the origins of social behavior. Curr. Opin. Insect. Sci..

[21] Lin S, Werle J, Korb J (2021). Transcriptomic analyses of the termite, Cryptotermes secundus, reveal a gene network underlying a long lifespan and high fecundity. Commun. Biol..

[22] Korb J (2015). A genomic comparison of two termites with different social complexity. Front. Genet..

[23] Korb J (2016). Genes underlying reproductive division of labor in termites, with comparisons to social Hymenoptera. Front. Ecol. Evol..

[24] Miura T, Maekawa K (2020). The making of the defensive caste: Physiology, development, and evolution of the soldier differentiation in termites. Evol. Dev..

[25] Rau, V. & Korb, J. The effect of environmental stress on ageing in a termite species with low social complexity. *Philos. Trans. R. Soc. Lond. B Biol. Sci.* **376**, 20190739 (2021). 10.1098/rstb.2019.0739 PMC7938165 33678015

[26] Inward D, Beccaloni G, Eggleton P (2007). Death of an order: A comprehensive molecular phylogenetic study confirms that termites are eusocial cockroaches. Biol. Lett..

[27] Abe, T. Evolution of Life Types in Termites (1987).

[28] Roisin Y, Korb J (2011). Social organisation and the status of workers in termites in Biology of Termites: A Modern Synthesis 133–164.

[29] Roisin Y (2000). Diversity and Evolution of Caste Patterns in Termites: Evolution, Sociality, Symbioses, Ecology 95–119.

[30] Korb J, Hartfelder K (2008). Life history and development—A framework for understanding developmental plasticity in lower termites. Biol. Rev. Camb. Philos. Soc..

[31] Rosengaus RB, Traniello JFA (1993). Temporal polyethism in incipient colonies of the primitive termite Zootermopsis angusticollis: A single multiage caste. J. Insect. Behav..

[32] Puig O, Marr MT, Ruhf ML, Tjian R (2003). Control of cell number by Drosophila FOXO: Downstream and feedback regulation of the insulin receptor pathway. Genes. Dev..

[33] Van Der Heide LP, Hoekman MFM, Smidt MP (2004). The ins and outs of FoxO shuttling: Mechanisms of FoxO translocation and transcriptional regulation. Biochem. J..

[34] Jongepier E (2018). Remodeling of the juvenile hormone pathway through caste-biased gene expression and positive selection along a gradient of termite eusociality. J. Exp. Zool. Mol. Dev. Evol..

[35] Helvig C, Unnithan GC (2004). CYP15A1, the cytochrome P450 that catalyzes epoxidation of methyl farnesoate to juvenile hormone III in cockroach corpora allata. Proc. Natl. Acad. Sci. USA.

[36] Weil T, Korb J, Rehli M (2009). Comparison of queen-specific gene expression in related lower termite species. Mol. Biol. Evol..

[37] Vesala L, Salminen TS, Kankare M, Hoikkala A (2012). Photoperiodic regulation of cold tolerance and expression levels of regucalcin gene in Drosophila montana. J. Insect. Physiol..

[38] Findlay GD, Yi X, MacCoss MJ, Swanson WJ (2008). Proteomics reveals novel drosophila seminal fluid proteins transferred at mating. PLoS Biol..

[39] Findlay GD, MacCoss MJ, Swanson WJ (2009). Proteomic discovery of previously unannotated, rapidly evolving seminal fluid genes in Drosophila. Genome. Res..

[40] Gramates, L. S. *et al.* FlyBase: A guided tour of highlighted features. *Genetics* **220**, (2022). 10.1093/genetics/iyac035 PMC8982030 35266522

[41] Qiu B (2022). Canalized gene expression during development mediates caste differentiation in ants. Nat. Ecol. Evol..

[42] Korb J, Heinze J (2016). Major hurdles for the evolution of sociality. Annu. Rev. Entomol..

[43] Terrapon N (2014). Molecular traces of alternative social organization in a termite genome. Nat. Commun..

[44] Weil T, Rehli M, Korb J (2007). Molecular basis for the reproductive division of labour in a lower termite. BMC Genom..

[45] Costa-Leonardo AM, Laranjo LT, Janei V, Haifig I (2013). The fat body of termites: Functions and stored materials. J. Insect. Physiol..

[46] Morandin C (2014). Not only for egg yolk—Functional and evolutionary insights from expression, selection, and structural analyses of formica ant vitellogenins. Mol. Biol. Evol..

[47] Yaguchi H (2023). Evolution and functionalization of vitellogenin genes in the termite Reticulitermes speratus. J. Exp. Zool. B Mol. Dev. Evol..

[48] Watanabe D, Gotoh H, Miura T, Maekawa K (2014). Social interactions affecting caste development through physiological actions in termites. Front. Physiol..

[49] Korb J (2015). Juvenile hormone: A central regulator of termite caste polyphenism. Adv. Insect. Phys..

[50] Partridge L, Alic N, Bjedov I, Piper MDW (2011). Ageing in Drosophila: The role of the insulin/Igf and TOR signalling network. Exp. Gerontol..

[51] Rau V, Flatt T, Korb J (2023). The remoulding of dietary effects on the fecundity / longevity trade-off in a social insect. BMC Genom..

[52] Flatt T, Tu MP, Tatar M (2005). Hormonal pleiotropy and the juvenile hormone regulation of Drosophila development and life history. BioEssays.

[53] Elsner D, Meusemann K, Korb J (2018). Longevity and transposon defense, the case of termite reproductives. Proc. Natl. Acad. Sci. USA.

[54] Monroy Kuhn JM, Meusemann K, Korb J (2019). Long live the queen, the king and the commoner? Transcript expression differences between old and young in the termite Cryptotermes secundus. PLoS ONE.

[55] Séité S (2022). Lifespan prolonging mechanisms and insulin upregulation without fat accumulation in long-lived reproductives of a higher termite. Commun. Biol..

[56] Monroy Kuhn JM, Meusemann K, Korb J (2021). Disentangling the aging gene expression network of termite queens. BMC Genom..

[57] de Souza Araujo N, Hellemans S, Roisin Y, Fournier D (2023). Transcriptomic profiling of castes and of sexually and parthenogenetically produced reproductive females in the termite Cavitermes tuberosus. Entomol. Exp. Appl..

[58] Cole EL, Ilieş I, Rosengaus RB (2018). Competing physiological demands during incipient colony foundation in a social insect: Consequences of pathogenic stress. Front. Ecol. Evol..

[59] Hartke TR, Rosengaus RB (2013). Costs of pleometrosis in a polygamous termite. Proc. R. Soc. B Biol. Sci..

[60] Rosengaus RB, Traniello JFA, Lefebvre ML, Carlock DM (2000). The social transmission of disease between adult male and female reproductives of the dampwood termite Zootermopsis angusticollis. Ethol. Ecol. Evol..

[61] Brent CS, Traniello JFA (2001). Social influence of larvae on ovarian maturation in primary and secondary reproductives of the dampwood termite Zootermopsis angusticollis. Physiol. Entomol..

[62] Nutting, W. L. Flight and Colony Foundation in *Biology of Termites* 233–282 (Elsevier, 1969).

[63] Rosengaus RB, Traniello JFA (1993). Disease risk as a cost of outbreeding in the termite Zootermopsis angusticollis. Proc. Natl. Acad. Sci. USA.

[64] Bolger AM, Lohse M, Usadel B (2014). Trimmomatic: A flexible trimmer for Illumina sequence data. Bioinformatics.

[65] Andrews, S. FastQC: A quality control tool for high throughput sequence data. Preprint at http://www.bioinformatics.babraham.ac.uk/projects/fastqc/ (2010).

[66] Altschul SF, Gish W, Miller W, Myers EW, Lipman DJ (1990). Basic local alignment search tool. J. Mol. Biol..

[67] Jones P (2014). InterProScan 5: Genome-scale protein function classification. Bioinformatics.

[68] Love MI, Huber W, Anders S (2014). Moderated estimation of fold change and dispersion for RNA-seq data with DESeq2. Genome. Biol..

[69] Benjamini Y, Hochberg Y (1995). Controlling the false discovery rate: A practical and powerful approach to multiple testing. J. R. Stat. Soc. B Stat..

[70] Zhang, B. & Horvath, S. A general framework for weighted gene co-expression network analysis. *Stat. Appl. Genet. Mol. Biol.* **4**, Article17. 10.2202/1544-6115.1128 (2005). 10.2202/1544-6115.1128 16646834

[71] Langfelder P, Horvath S (2008). WGCNA: An R package for weighted correlation network analysis. BMC Bioinform..

[72] Alexa, A. & Rahnenfuhrer, J. topGO: Enrichment Analysis for Gene Ontology. (2016).

[73] Harrison MC (2018). Hemimetabolous genomes reveal molecular basis of termite eusociality. Nat. Ecol. Evol..

