# Supplementary material for: A genetic toolkit underlying the queen phenotype in termites with totipotent workers
Source: Sci Rep. 2024 Jan 26;14:2214. doi: 10.1038/s41598-024-51772-7 (PMC10817970; doi:10.1038/s41598-024-51772-7)
Supplement: Supplementary file 1 — Supplementary Information. [file 41598_2024_51772_MOESM1_ESM.pdf]

Supplemental Information for:

A genetic toolkit underlying the queen phenotype in termites with totipotent workers

Silu Lin, Daniel Elsner, Leon Ams, Judith Korb, Rebeca Rosengaus

Table S1. Soft-threshold powers and quality control for WGCNA analysis.

Analysis	Soft-threshold power	Number of genes removed before WGCNA analysis
Head: queen vs worker	18	1633
Abdomen: queen vs worker	15	1551
Head: worker vs early larvae	15	1655
Abdomen: worker vs early larvae	16	1659

Table S2. Results of the QCM enrichment analyses for DEGs.

Analysis	Result	Nr. of genes	Nr. of QCM homologues	Odds ratio	P value
Head: queens vs workers	Upregulated in queens	251	16	3.803	<0.001
	Upregulated in workers	479	4	0.441	0.117
Abdomen: queens vs workers	Upregulated in queens	2653	29	0.543	0.001
	Upregulated in workers	2715	77	1.803	<0.001
Head: workers vs larvae	Upregulated in workers	160	12	4.478	<0.001
	Upregulated in larvae	34	1	1.616	0.469
Abdomen: workers vs larvae	Upregulated in workers	118	16	8.830	<0.001
	Upregulated in larvae	62	0	0	0.632

In Table S3, there should be a line between darkred and yellowgreeen to seperate the modules between head and abdomen tissues.

Table S3. Enrichment of QCM genes in the modules that characterize queens compared to workers (i.e., queen modules).

Analysis	Module	Nr. genes	Nr. QCM homologues	Odds ratio	P value
Head	*red*	352	18	3.014	<0.001
	darkolivegreen	135	11	4.896	<0.001
	turquoise	665	28	2.506	<0.001
	salmon4	90	4	2.505	0.084
	darkgrey	178	4	1.230	0.572
	lightcyan1	115	3	1.434	0.472
	darkred	189	9	2.727	0.008
Abdomen	*yellowgreen*	130	1	0.414	0.734
	blue	757	12	0.958	1.000
	darkgreen	189	0	0	0.053
	turquoise	861	11	0.681	0.239
	royalblue	201	2	0.536	0.592
	thistle2	92	0	0	0.419
	darkslateblue	98	2	1.119	0.701
	lightcoral	60	3	2.846	0.097
	brown2	48	1	1.143	0.588
	violet	140	1	0.384	0.525
	honeydew1	80	0	0	0.408
	bisque4	99	0	0	0.267
	orange	177	2	0.610	0.775
	darkred	192	5	1.444	0.406
	maroon	82	0	0	0.409
	yellow4	73	0	0	0.409

Table S4. Enrichment of QCM genes in the modules that characterize workers compared to queens.

Analysis	Module	Nr. genes	Nr. QCM homologues	Odds ratio	P value
Head	*lightcyan*	248	2	0.430	0.335
	darkslateblue	99	0	0	0.268
	midnightblue	269	3	0.597	0.495
	violet	147	2	0.734	1.000
	lightyellow	215	3	0.752	1.000
	yellow4	67	4	3.425	0.035
	green	367	8	1.195	0.554
	lightpink3	42	0	0	1.000
	floralwhite	107	3	1.545	0.451
Abdomen	*skyblue2*	74	0	0	0.648
	midnightblue	299	32	7.188	<0.001
	brown	578	30	3.189	<0.001
	darkorange	171	5	1.629	0.246
	yellow	493	18	2.110	0.005
	lightyellow	211	6	1.584	0.289
	salmon	325	14	2.496	0.003
	lightcyan	265	2	0.404	0.247
	black	397	4	0.539	0.337
	lightgreen	211	1	0.253	0.192

Table S5. Enrichment of QCM genes in the modules that characterize larvae compared to workers (larvae-specific modules).

Analysis	Module	Nr. genes	Nr. QCM homologues	Odds ratio	P value
Head	*green*	550	4	0.381	0.050
	thistle3	41	0	0	1.000
Abdomen	*cyan*	250	1	0.211	0.095
	palevioletred2	40	1	1.367	0.525

Table S6. Enrichment of QCM genes in the modules that characterize workers compared to larvae.

Analysis	Module	Nr. genes	Nr. QCM homologues	Odds ratio	P value
Head	*paleturquoise*	129	10	4.614	0.0001
	salmon4	85	5	3.375	0.020
	turquoise	858	38	2.718	<0.001
	darkorange2	102	7	4.005	0.003
Abdomen	*black*	332	13	2.232	0.011

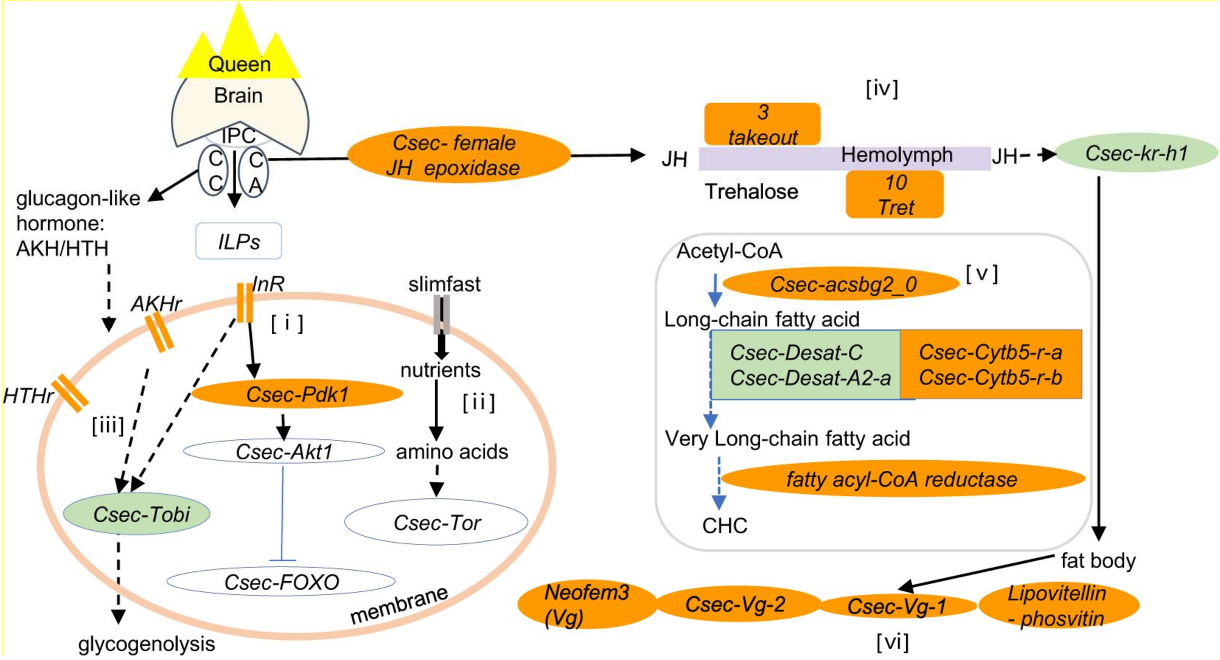

Figure S1. Schematic representation of the queen central module (QCM) (After[1]). The figure illustrates genes co-expressed in the QCM (orange) and other queen modules (greenish) within nutrient-sensing pathways, including (i) IIS and (ii) TOR, (iii) neuro-hormonal regulation of metabolic processes, (iv) JH signaling (including trehalose transport) in the upper part, and (v) CHC production and (vi) vitellogenesis. The left section focuses on gene regulatory pathways related to neuroendocrine and IIS/TOR signaling, while the right section emphasizes fecundity- and CHC-related processes. Both sections are connected by hemolymph transport processes. Genes are represented by circles/double bars, gene 'clusters' by squares, activation by arrows, and repression by stop bars. Solid and dashed lines indicate direct and supposed indirect interactions, respectively, requiring further investigation. Abbreviations: IPC (insulin-producing cells), CC (corpus cardiacum), CA (corpora allata).

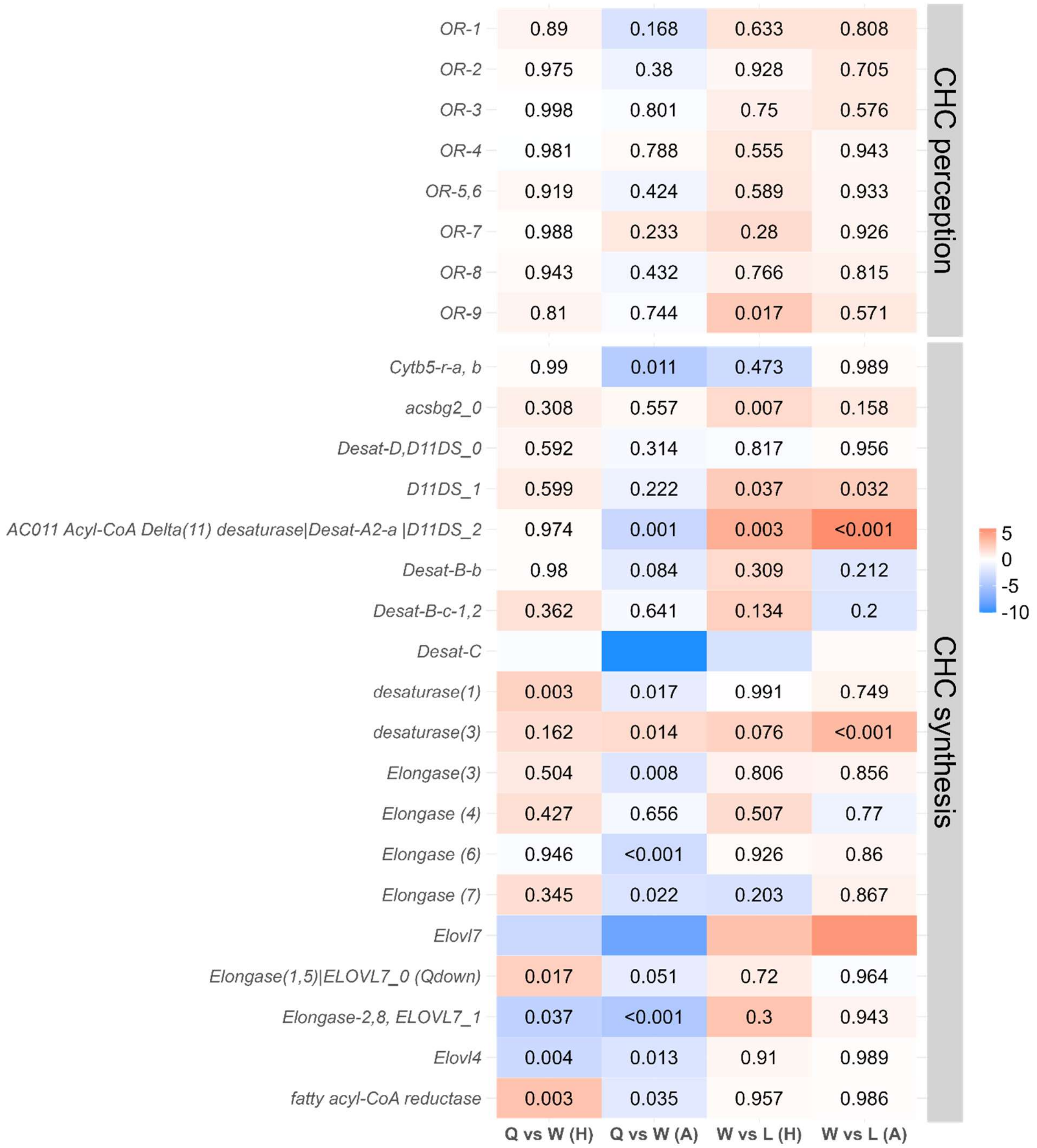

Figure S2. Results of the differentially expressed gene analyses for CHC-related genes. Each row represents a gene. Column 1 shows the results for the queen-worker comparison of head samples and Column 2 those of the abdomen samples. Column 3 and 4 represent the results for the worker-larvae comparisons for head and abdomens, respectively. The color bar reflects log2 fold change (LFC) in gene expression with red indicating a higher expression of the first compared to second group (e.g., in column 1, a higher expression in queens than workers) and blue *vice versa*. The value in each cell shows the adjusted *P* value. Abbreviations: Q = queens, W= workers, H = heads, A = abdomens, L = Larvae.

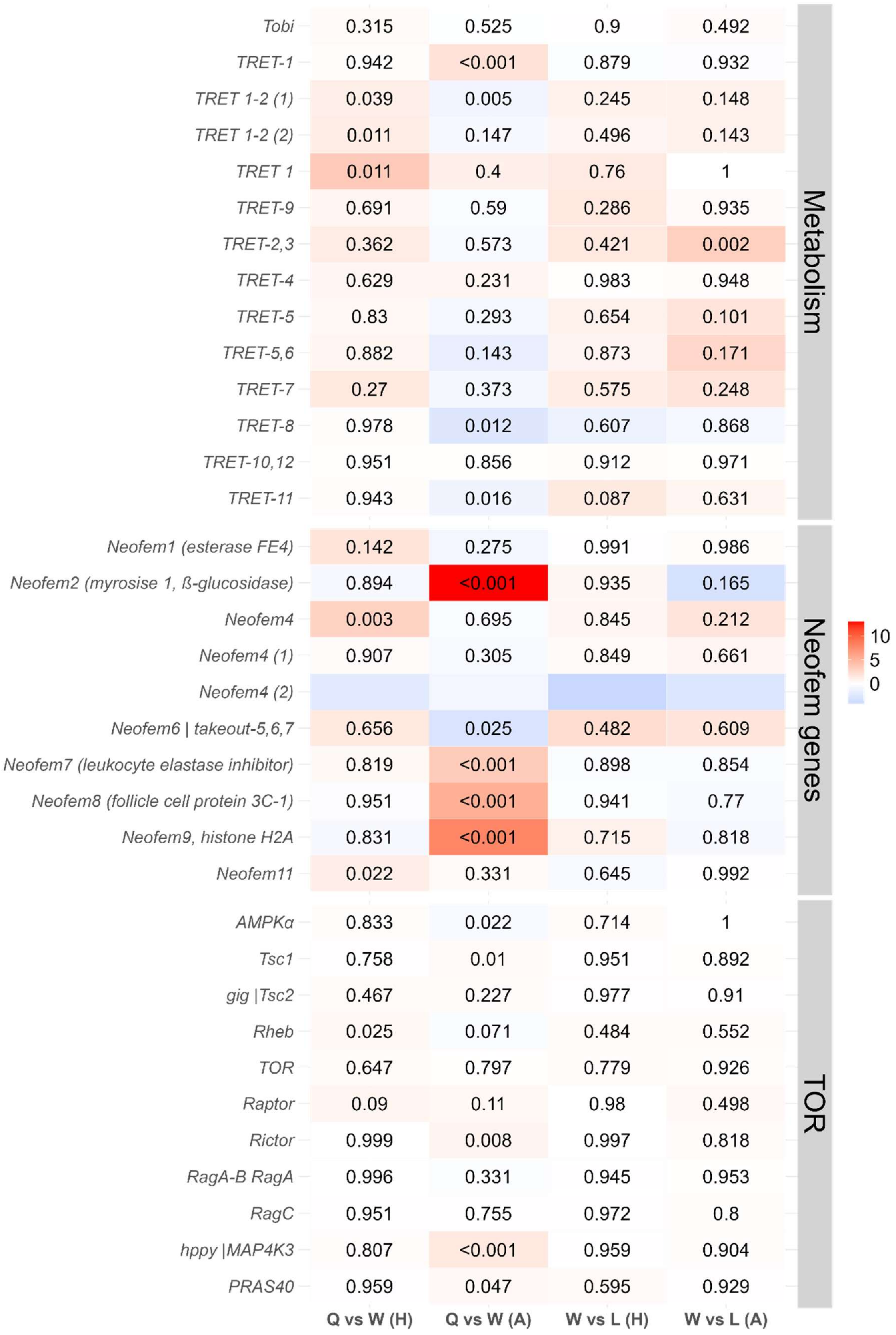
Tobi 0.315 0.525 0.9 0.492
TRET-1 0.942 <0.001 0.879 0.932
TRET 1-2 (1) 0.039 0.005 0.245 0.148
TRET 1-2 (2) 0.011 0.147 0.496 0.143
TRET 1 0.011 0.4 0.76 1
TRET-9 0.691 0.59 0.286 0.935
TRET-2,3 0.362 0.573 0.421 0.002
TRET-4 0.629 0.231 0.983 0.948
TRET-5 0.83 0.293 0.654 0.101
TRET-5,6 0.882 0.143 0.873 0.171
TRET-7 0.27 0.373 0.575 0.248
TRET-8 0.978 0.012 0.607 0.868
TRET-10,12 0.951 0.856 0.912 0.971
TRET-11 0.943 0.016 0.087 0.631
Metabolism
Neofem1 (esterase FE4) 0.142 0.275 0.991 0.986
Neofem2 (myrosise 1, ß-glucosidase) 0.894 <0.001 0.935 0.165
Neofem4 0.003 0.695 0.845 0.212
Neofem4 (1) 0.907 0.305 0.849 0.661
Neofem4 (2)
Neofem6 | takeout-5,6,7 0.656 0.025 0.482 0.609
Neofem7 (leukocyte elastase inhibitor) 0.819 <0.001 0.898 0.854
Neofem8 (follicle cell protein 3C-1) 0.951 <0.001 0.941 0.77
Neofem9, histone H2A 0.831 <0.001 0.715 0.818
Neofem11 0.022 0.331 0.645 0.992
Neofem genes
AMPKα 0.833 0.022 0.714 1
Tsc1 0.758 0.01 0.951 0.892
gig |Tsc2 0.467 0.227 0.977 0.91
Rheb 0.025 0.071 0.484 0.552
TOR 0.647 0.797 0.779 0.926
Raptor 0.09 0.11 0.98 0.498
Rictor 0.999 0.008 0.997 0.818
RagA-B RagA 0.996 0.331 0.945 0.953
RagC 0.951 0.755 0.972 0.8
hppy |MAP4K3 0.807 <0.001 0.959 0.904
PRAS40 0.959 0.047 0.595 0.929
TOR
Q vs W (H)
Q vs W (A)
W vs L (H)
W vs L (A)
10
5
0

Figure S3. Results of the differentially expressed gene analyses for metabolism-related genes, Neofem genes, and genes of the TOR (target of rapamycin) pathway. Each row represents a gene. Column 1 shows the results for the queen-worker comparison of head samples and Column 2 those of the abdomen samples. Column 3 and 4 represent the results for the worker-larvae comparisons for head and abdomens, respectively. The color bar reflects log2 fold change (LFC) in gene expression with red indicating a higher expression of the first compared to second group (e.g., in column 1, a higher expression in queens than workers) and blue vice versa. The value in each cell shows the adjusted *P* value. Abbreviations: Q = queens, W= workers, H = heads, A = abdomens, L = Larvae.

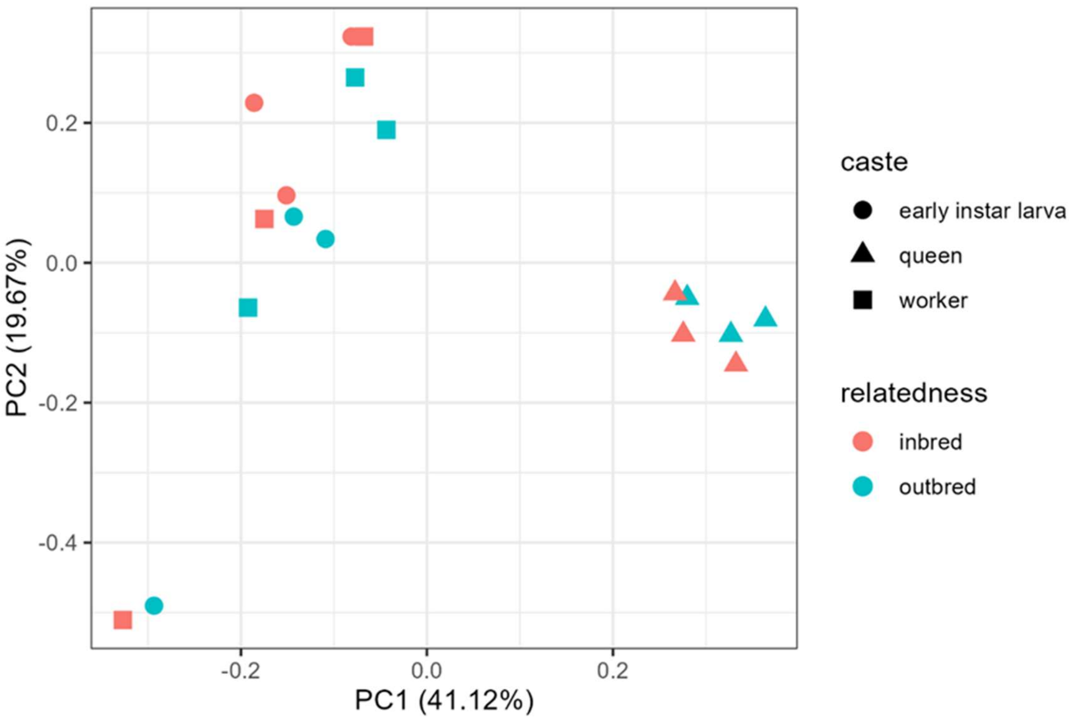

Figure S4: Results of a principal component analysis (PCA) for abdomens (without guts) using the 500 genes with the largest variance. Principle component (PC) 1 and 2 explain 41.1% and 19.7% of the variance, respectively. Different castes are indicated by different shapes. PC1 separates queens from all other castes.; early instar larvae and workers were not clearly separated.

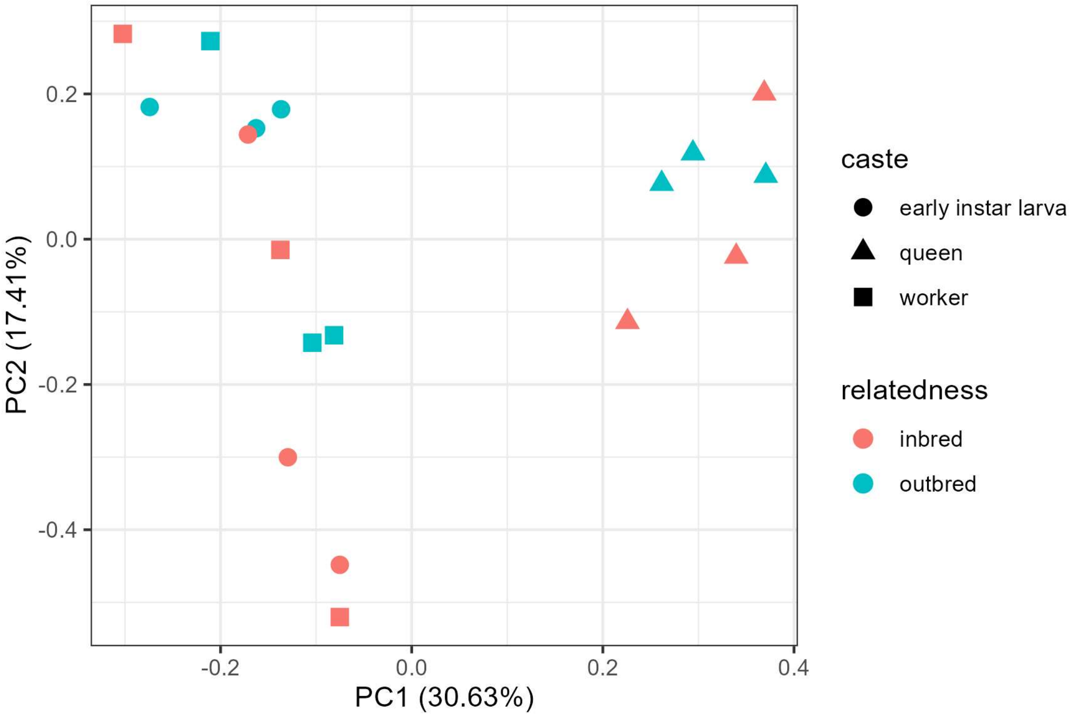

Figure S5. Results of a principal component analysis (PCA) for abdomens (without guts) using all genes. Principle component (PC) 1 and 2 explain 30.6% and 17.4% of the variance, respectively. Different castes are indicated by different shapes. Similar to Figure S1, PC1 separates queens from all other castes.

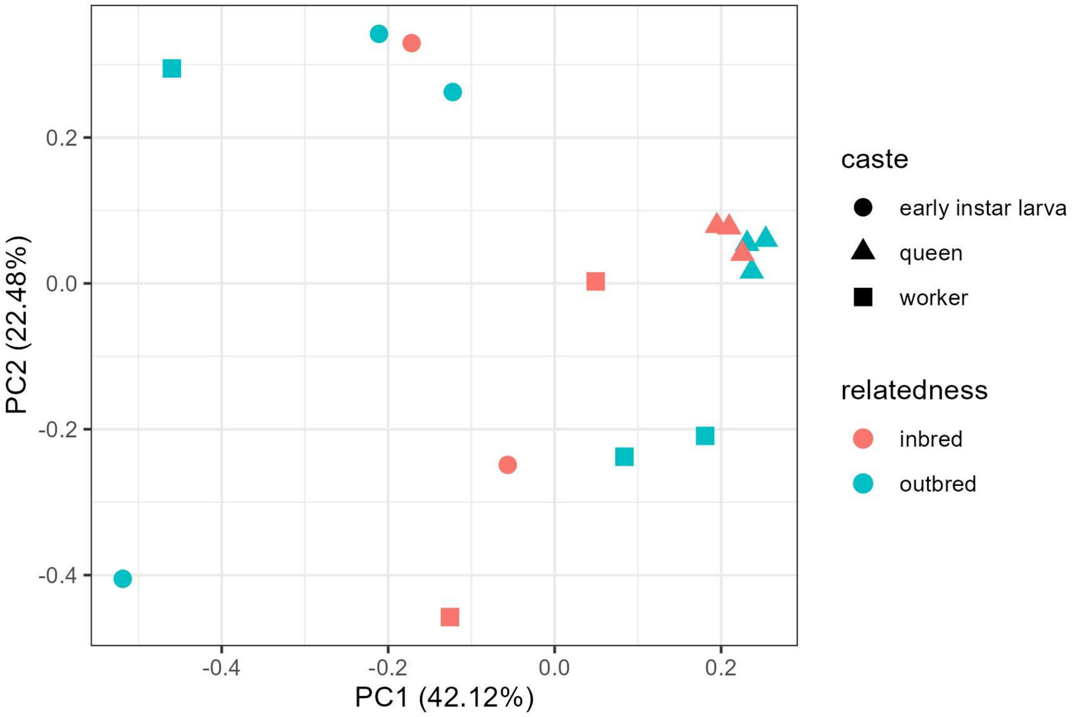

Figure S6: Results of a principal component analysis (PCA) for heads (with prothorax) using the 500 genes with the largest variance. PC1 and PC2 explain 42.1% and 22.5% of the variance, respectively. Castes are represented by different shapes. PCA1 separates early instar larvae and workers, and also queens and workers.

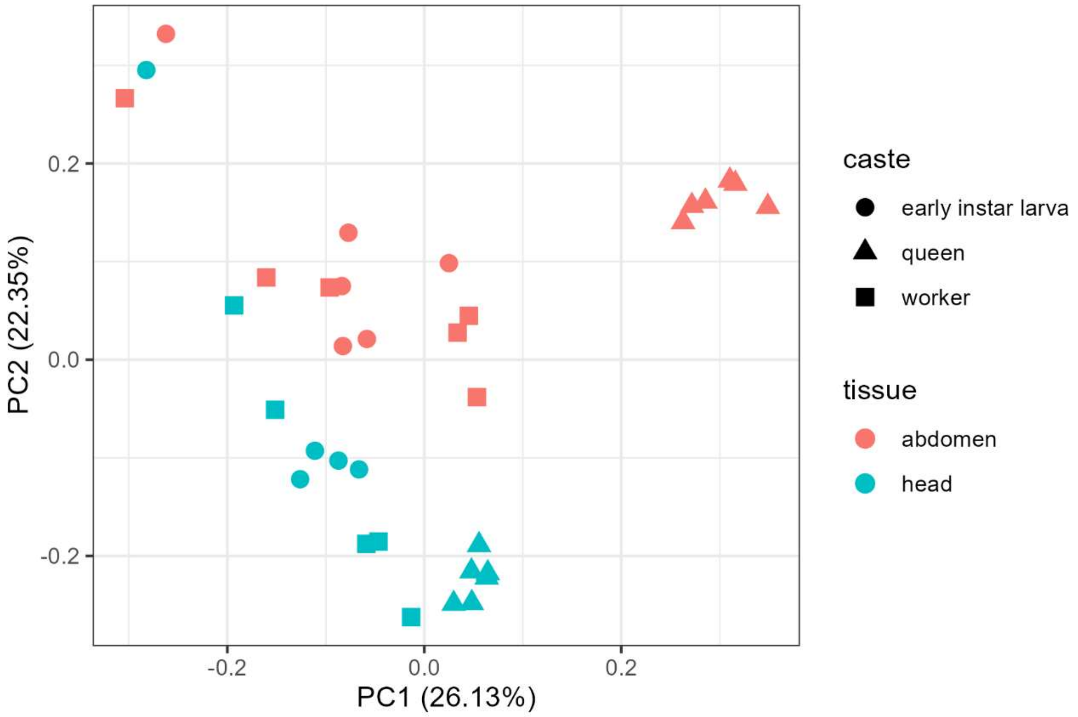

Figure S7: Results of a PCA of the 500 genes with the greatest variance for samples from both tissues. Shown are PC1 and PC2, which account for 26.1% and 22.4% of the variance, respectively. Heads (plus prothorax) are shown blue and abdomens (without gut) in red. Shapes represent different castes. Abdomen and head tissues were separated mainly by PC2. Queens were separated by PC1. There was no clear separation between workers and early instar larvae.

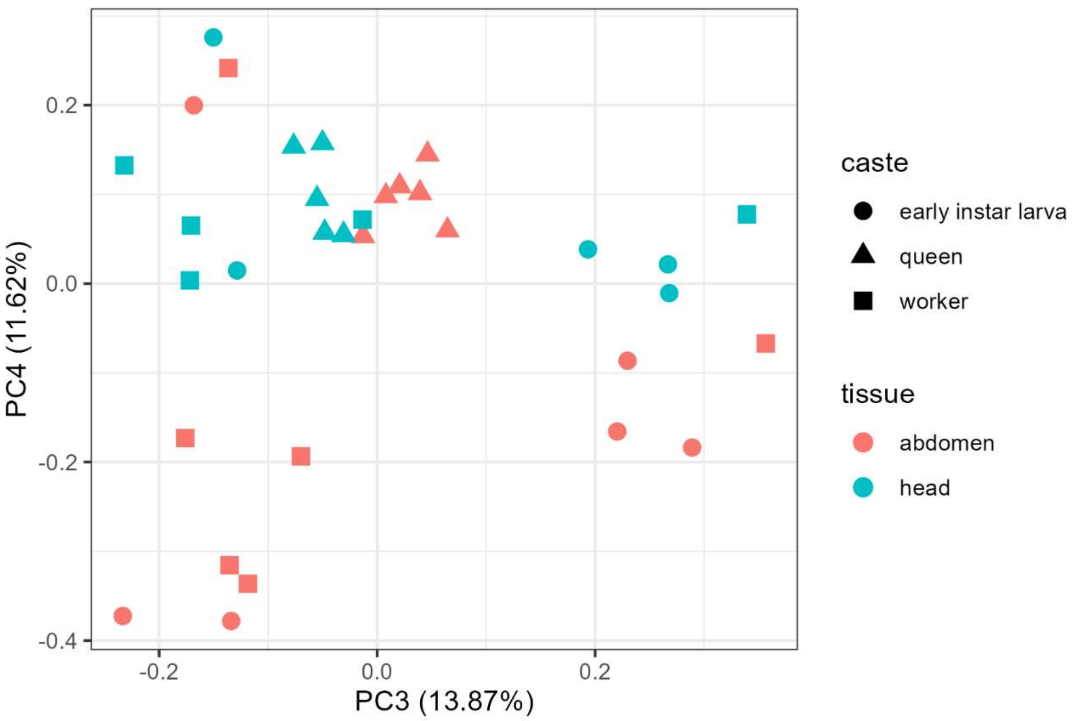

Figure S8: Results of a PCA of the 500 genes with the greatest variance for samples from both tissues. Shown are PC3 and PC4. PC3 and PC4 explain 13.9% and 11.6% of the variance, respectively. Heads (plus prothorax) are shown blue and abdomens (without gut) in red. Shapes represent different castes. There is no clear separation by caste or tissue.

Results of GO enrichment analysis on DEGs

Queens vs. workers

For the genes that were upregulated in queen compared to worker heads, the enriched GO terms were: transmembrane transport (BP: GO:0055085), lipid transport (BP: GO:0055085), extracellular space (CC: GO:0005615), lipid transporter activity (MF: GO:0005319), and iron ion binding (MF: GO:0005506).

For the genes that were upregulated in queen compared to worker abdomens, the enriched GO terms were: DNA repair (BP), nucleus (CC: GO:00056…34), chromosome (CC: GO:0005694), nucleosome (CC: GO:0000786), protein binding (MF: GO:0005515), and DNA binding (MF: GO:0003677).

For the genes that were upregulated in worker compared to queen heads, the enriched GO terms were: proteolysis (BP: GO:0000786), DNA replication initiation (BP: GO:0006270), extracellular region (CC: GO:0005576), collagen trimer (CC: GO:0005581), extracellular matrix (MF: GO:0005201) and chitin binding (MF: GO:0008061).

For the genes that were upregulated in worker compared to queen abdomens, the enriched GO terms were: transmembrane transport (BP: GO:0055085), proteolysis (GO:0006508), extracellular region (CC: GO:0005576), membrane (CC: GO:0016020), integral component of membrane (CC: GO:0016021), chitin binding (MF: GO:0008061), and calcium ion binding (GO:0005509).

Workers vs. early instar larvae

For the genes that were upregulated in worker heads compared to the heads of early instar larvae, the enriched GO terms were: superoxide metabolic process (BP: GO:0006801), transmembrane transport (BP: GO:0055085), extracellular region (CC: GO:0005576), junctional membrane complex (CC: GO:0030314), pyridoxal phosphate binding (MF: GO:0030170) and iron ion binding (MF: GO:0005506).

For the genes that were upregulated in worker abdomens compared to the abdomens of early instar larvae, the enriched GO terms were: lipid metabolic process (BP: GO:0006629), proteolysis (BP: GO:0006508), extracellular region (CC: GO:0005576), extracellular space (CC: GO:0005615), monooxygenase activity (MF: GO:0004497) and heme binding (MF: GO:0020037).

For the genes that were upregulated in the heads of early instar larvae compared to worker heads, the enriched GO terms were: rRNA catabolic process (BP: GO:0016075), pre−miRNA processing (BP: GO:0031054), integral component of membrane (CC: GO:0016021), protein domain specific binding (MF: GO:0019904) and ribonuclease III activity (MF: GO:0004525).

For the genes that were upregulated in the abdomens of early instar larvae compared to worker abdomens, the enriched GO terms were: protein autoprocessing (BP: GO:0016540), arginyl-tRNA aminoacylation (BP: GO:0006420), Prp19 complex (CC: GO:0000974), nucleosome (CC: GO:0000786), DNA binding (MF: GO:0003677), and RNA binding (MF: GO:0003723).

Overview of the supplementary data

Data S1. Details of samples.
Data S2. Annotations of the *Z. nevadensis* genome.
Data S3. DEG results for the queen-worker comparison.
Data S4. DEG results for the worker-larvae comparison.

Data S5. 16 QCM homologues among the DEGs that characterize queen heads.
Data S6. Modules that were specific of worker heads, compared to queens.
Data S7. Modules that were specific of queen heads, compared to workers.
Data S8. Modules that were specific of worker abdomens, compared to queens.
Data S9. Modules that were specific of queen abdomens, compared to workers.
Data S10. Modules that were specific of larvae heads, compared to workers.
Data S11. Modules that were specific of worker heads, compared to larvae.
Data S12. Modules that were specific of larvae abdomens, compared to workers.
Data S13. Modules that were specific of worker abdomens, compared to larvae.

Reference

1. Lin, S., Werle, J. & Korb, J. Transcriptomic analyses of the termite, Cryptotermes secundus, reveal a gene network underlying a long lifespan and high fecundity. *Commun. Biol.* **4**, 384; https://doi.org/10.1038/s42003-021-01892-x (2021).
